# Active Peptides from Crayfish Shell: Isolation, Purification, Identification and Cytoprotective Function on Cells Damaged by H_2_O_2_

**DOI:** 10.3390/biom15091225

**Published:** 2025-08-26

**Authors:** Chan Bai, Wenqing Wang, Guowei Huang, Ya Wang, Xiaoyan Zu, Liang Qiu, Ziyi Tu, Wei Yu, Tao Liao

**Affiliations:** 1 Key Laboratory of Cold Chain Logistics Technology for Agro-Product, Ministry of Agriculture and Rural Affairs, Institute of Agro-Product Processing and Nuclear Agricultural Technology, Hubei Academy of Agricultural Sciences, No.5 Nanhu Avenue, Hongshan District, Wuhan 430064, Chinawangwenq@lut.edu.cn (W.W.); gwhuang@mail.hbut.edu.cn (G.H.); zuxiaoyan@hbaas.ac.cn (X.Z.);; 2 School of Life Science and Engineering, Lanzhou University of Technology, Lanzhou 730050, China; wangya502@lut.edu.cn; 3 School of Life Sciences and Health Engineering, Hubei University of Technology, Wuhan 430068, China; 4 Hubei Crayfish Industrial Technology Research Institute Co., Ltd., Qianjiang 433115, China

**Keywords:** crayfish shell peptide, biological activity, mass spectrometry identification, cytoprotective effect

## Abstract

This study presents a strategy to develop crayfish shell peptides with enhanced antioxidant and angiotensin-I-converting enzyme (ACE) inhibitory properties. Crayfish shell protein hydrolysates (CSPH1–3) with different molecular weights were analyzed. CSPH2 (3–5 kDa) exhibited the strongest antioxidant activities, which could scavenge 1,1-diphenyl-2-picrylhydrazyl (DPPH) and the 2,2′-azobis(3-ethylbenzothiazoline-6-sulfonic acid) sodium salt (ABTS) radical by (77.40 ± 4.54)% and (91.59 ± 0.30)%, respectively, and ACE inhibition activity of (64.74 ± 0.64)%. CSPH2 was further separated into three fractions, and CSPHF2 showed the maximum biological activity. The sequences of the purified antioxidant peptide (APAPLPPPAP) and ACE inhibitory peptide (QGPDDPLIPIM) were identified by liquid chromatography–tandem mass spectrometry (LC-MS/MS) in CSPHF2. These peptides increased the nitric oxide (NO) concentration and decreased the endothelin-1 (ET-1) content in human umbilical vein endothelial cells (HUVECs) in a dose-dependent manner, while also inhibiting reactive oxygen species (ROS). In addition, CSPH showed protective effects in terms of oxidative damage to HepG2 cells induced by H_2_O_2_. These findings suggest that crayfish shell peptides have potential applications as ingredients in antihypertensive agents and antioxidants, offering significant health benefits when consumed.

## 1. Introduction

*Procambarus clarkii*, commonly known as crayfish, is one of the most important farmed freshwater crayfish species globally [1,2]. China has seen a consistent upward trend in both the aquaculture area and the output of crayfish, with production reaching 3.16 million tons in 2023, accounting for 9.26% of the country’s total freshwater aquaculture production [3]. The processing volume also reached 850,000 tons. Crayfish have become a popular local food in China due to their good taste and high nutritional value. However, approximately 40% of crayfish end up as by-products (heads, shells, and tails), which are typically discarded as waste. This not only leads to environmental pollution and resource wastage but also incurs economic costs. Developing functionalized hydrolysates or peptides from these by-products could be a valuable strategy to address these issues. These by-products contain about 20–30% protein (dry weight), from which value-added compounds can be obtained. Proteins from aquatic organisms often have distinct sequences and strong biological functions, enabling them to thrive in demanding environments. As a result, there has been growing interest in bioactive peptides derived from aquatic sources, particularly those with antioxidant and angiotensin-converting enzyme (ACE) inhibitory properties [4,5].

The combination of ACE inhibition and antioxidant activity in a single product could be highly beneficial for controlling cardiovascular diseases [6]. In recent decades, the recovery of protein hydrolysates from various marine organisms and their by-products has gained significant attention. Examples include scallop offal [7], grass carp scales [8], and Australian rock lobster shells [9]. These hydrolysates typically contain a mixture of peptides with different molecular weights and free amino acids, exhibiting various bioactivities, such as antioxidant [10], antihypertensive [11,12], anti-inflammatory [13], and antimicrobial activities. Despite the popularity of protein hydrolysis from animal sources due to their bioaccessibility and safety, the potential of crayfish shell proteins for developing hydrolysates remains largely unexplored.

While many studies have focused on the functionality of peptides, few have considered their applications in functional foods or pharmaceuticals. To further utilize bioactive peptides in practical applications, their cellular mechanisms of action need to be elucidated. For instance, Hu et al. isolated antioxidant peptides (EDIVCW and YWDAW) from the muscle hydrolysate of *Ankangus auratus* and investigated their protective mechanisms against oxidative damage in human hepatocarcinoma cells (HepG2) through changes in the reactive oxygen species (ROS) content [8]. Similarly, Chen et al. studied the ACE inhibitory peptide (VISDEDGVTH) from *Bacillus natto* fermentum and explored its cellular mechanism in human umbilical vein cells (HUVECs) [14]. However, the cellular mechanisms of bioactivity for crayfish shell peptides have not been fully investigated, which is a necessary prerequisite for understanding their potential toxicity risks or cytoprotective functions.

Therefore, the objective of this study is to isolate, purify, and identify bioactive peptides with antioxidant and ACE inhibitory properties from crayfish shells. We aim to further investigate the mechanisms underlying the antioxidant and ACE inhibitory activities of these peptides at the cellular level and their cytoprotective effects on H_2_O_2_-damaged HUVECs. This research will not only explore the potential food and pharmaceutical functions of crayfish shell peptides but also provide a theoretical framework for the development of novel antioxidants and ACE inhibitors, facilitating the reuse of crayfish by-products.

## 2. Materials and Methods

### 2.1. Materials

Crayfish shells was kindly supplied by Wuhan Liangzihu Aquatic Products Processing Co., Ltd. (Wuhan, China) Nitric oxide (NO), the Endothelin-1 (ET-1) and Reactive Oxygen Species (ROS) Kit, Sephadex G-25, artificial gastric fluid, and artificial intestinal fluid were purchased from Shanghai Yuanye Biotechnology Co., Ltd. (Shanghai, China). Fetal bovine serum, minimum essential medium (MEM), 3-(4,5-dimethyl-2-thiazolyl)-2,5-diphenyl-2-H-tetrazolium bromide (MTT), phosphate-buffered saline (PBS), and dimethyl sulfoxide (DMSO) were purchased from Beijing Solaibao Technology Co., Ltd. (Beijing, China) 1,1-diphenyl-2-picrylhydrazyl (DPPH), 2,2′-azinobis-(3-ethylbenzthiazoline-6-sulphonate(ABTS) and ACE were purchased from Shanghai Maclean Biochemical Technology Co., Ltd. (Shanghai, China). The HepG2 (HUVEC) cell line was purchased from Shanghai Saibakang Biological Co., Ltd. (Shanghai, China).

### 2.2. Preparation of Crayfish Shell Protein Hydrolysate (CSPH)

CSPH was prepared in the laboratory. The enzymatic hydrolysis conditions for the CPSH was chosen on the basis of earlier studies using response surface methodology: combined enzyme of alkaline protease and trypsin with an amount of 10,200 U/g and combine ration of 13:20; enzymatic digestion time of 2.8 h and ultrasound time of 30 min. The prepared CSPH was freeze-dried and stored at −20 °C.

### 2.3. Peptide Separation via Ultrafiltration and Gel Permeation Chromatograph

Determination of the molecular weight distribution of CSPH was carried out by gel permeation chromatograph (GPC). The chromatographic conditions were as follows: column: 2× PLgel 8 μm aquagel-OH Mixed-M 7.5 × 300 mm; mobile phase: 0.02 mol/L KH2PO4; sample volume: 100 μL; flow rate:1.0 mL/min; column temperature: 40 °C; injection volume: 100 μL; standard: Agilent PEO/PEG (molecular weights: 202,400, 134,300, 72,750, 31,440, 3870, 615).

The lyophilized CSPH was dissolved in deionized water and the mixture were passed through two ultrafiltration membranes with molecular weight (MW) cut-offs of 5 kDa and 3 kDa, respectively. Three fractions with different molecular weight were obtained and named as CSPH1 (>5 kDa), CSPH2 (3–5 kDa) and CSPH3 (<3 kDa). Three fractions were freeze-dried and stored at −20 °C for further analysis. The bioactivity, including the antioxidant ability and ACE inhibitory activity of each fraction, was tested.

### 2.4. Bioactivity of Active Peptides

#### 2.4.1. Antioxidant Activity

The DPPH scavenging activity of the samples was determined using the method described by Jemil I et al. with slight modifications [15]. The DPPH radical scavenging ability (DSA) was calculated according to Equation (1).

(1) $$DAS\;(\%)=\frac{1-(A_{1}-A_{1j})}{{A_{1}}^{\prime }}\times 100\%$$

The absorbance in Equation (1) were measured at 517 nm, where A_1_ is the absorbance of the specimen, A_1_′ is the absorbance of the blank control, and A_1j_ is the absorbance of the sample background absorption correction. The EC_50_ values were defined as the concentration of samples that decrease the initial radical concentration of 50%.

The ABTS scavenging activity of the samples was determined using the method described by Zhang et al. with slight modifications [16]. The ABTS radical scavenging ability (ASA) was calculated according to Equation (2).

(2) $$ASA\;(\%)=\frac{1-(A_{2}-A_{2j})}{{A_{2}}^{\prime }}\times 100\%$$

The absorbance in Equation (2) was measured at 420 nm, where A_2_ was the absorbance of the sample, A_2_′ was the absorbance of the blank control, and A_2j_ was the absorbance of the sample corrected for background absorption. The EC_50_ values were defined as the concentration of samples that decrease the initial radical concentration of 50%.

#### 2.4.2. ACE Inhibitory Activity

The ACE inhibition rate was determined by the FAPGG method according to the method of Geng et al. with slight modifications [17]. The ACE inhibition rate was calculated according to Equation (3).

(3) $$\mathrm{ACE}\;\mathrm{inhibition}\;\mathrm{activity}(\%)=(1-\frac{∆A_{a}}{∆A_{b}})\times 100\%$$

In Equation (3), ΔA_a_ is the change in absorbance at 340 nm over 30 min after the addition of inhibitor and ΔA_b_ is the change in absorbance at 340 nm over 30 min after the addition of buffer. The half maximal inhibitory concentration (IC_50_) values of the peptides were defined as the concentration needed to inhibit activity to 50%.

### 2.5. Determination of Factors Affecting the Stability of CSPH2

The most active fraction after ultrafiltration separation was identified as CSPH2, and the stability of CSPH2 under different temperatures, pHs and simulation of the gastrointestinal tract environment was studied.

#### 2.5.1. Temperature

CSPH2 (500 μL of 1.0 mg/mL) was placed in a water bath at 25, 37, 50, 60 and 70 °C for 2 h, and then the DPPH scavenging and ACE inhibition were measured by the DPPH method and FAPGG method, respectively.

#### 2.5.2. pH

CSPH2 (200 μL of 10.0 mg/mL) was incubated at room temperature for 2 h at pH 2.0, 4.0, 6.0, 8.0, and 10.0, diluted ten times to the sample concentration of 1.0 mg/mL, and then the DPPH scavenging and ACE inhibition were measured by the DPPH method and FAPGG method, respectively.

#### 2.5.3. Simulation of Gastrointestinal Tract Environment In Vitro

The stability of CSPH2 in a simulated gastrointestinal digestion tract environment was investigated by the method described previously [18,19]. CSPH2 (200 μL of 10.0 mg/mL) was added to artificial gastric juice and artificial intestinal juice at 1:9 (*v*/*v*) [18], and placed in a 37 °C water bath. After incubation for 0, 30, 60, 90 and 120 min, the solutions was separately diluted 10 times, and the DPPH scavenging and the ACE inhibition rate were measured as mentioned above.

### 2.6. Low-Pressure Gel Filtration Chromatography

A 1.6 cm × 120 cm column was fixed vertically on the support. The Sephadex G-25 was inserted into a suction filter bottle and degassed under vacuum for 20 min. The excess liquid was discarded. The sedimentation medium and supernatant (*v*/*v* = 3:1) were used for chromatography. The column was equilibrated with 2 to 3 column volumes of eluent, and then the sample was loaded. CSPH2’s separated components were taken at 1 mg/mL, filtered through a 0.45 μm microporous filter, and loaded with the sample. Deionized water was used to elute at a certain flow rate. Fractions were collected every 3 min using an automatic collector and measured at 280 nm at room temperature. The three collected fractions, CSPHF1, CSPHF2, and CSPHF3, were lyophilized and stored at −20 °C before the ACE inhibitory activity and antioxidant activity were determined

### 2.7. Amino Acid Sequence Analysis of the Purified Peptide

The fraction with bioactive activity was analyzed using liquid chromatography–tandem mass spectrometry (LC-MS/MS) utilizing an Q Exactive mass spectrometer (Thermo Fisher, Waltham, MA, USA) device and an HPLC system (Agilent Technologies, Wilmington, DE, USA). The HPLC mobile phase consisted of the following solutions: liquid A: 0.1% formic acid and liquid B: 0.1% formic acid acetonitrile (84% acetonitrile). The liquid chromatography column (0.15 mm × 150 mm, RP-C18, Column Technology Inc., Lombard, IL, USA) was equilibrated with 95% of liquid A. Samples were loaded by autosampler onto Zorbax 300SB-C18 peptide traps (Agilent Technologies, Wilmington, DE, USA) and elution was performed as follows: 0–50 min 4–50% liquid B; 50–54 min 50–100% liquid B; 54–60 min 100% liquid B. The sample loading volume was 5 μL and the flow rate was 0.3 mL/min. The capillary temperature and ionization voltage were set to be 200 °C and 2.2 kVD. Duration of analysis: 60 min; detection: positive ions. The mass-to-charge ratios of the peptides and fragments of peptides were collected according to the following method: 10 fragment profiles (MS2 scan) after each full scan. The obtained peptides were matched by NCBI and identified for activity on the website PeptideRanker (http://distilldeep.ucd.ie/PeptideRanker). Only those peptides that presented “de novo” scores above 60% were considered for further analysis in this study. The biological activity potential of the peptides was determined using PeptideRanker (http://distilldeep.ucd.ie/PeptideRanker, accessed on 16 September 2024). The probability of the peptide having bioactivity was predicted by PeptideRanker and combined structure–activity relationship analysis, available at http://distilldeep.ucd.ie/PeptideRanker/, accessed on 16 September 2024, where the higher the score, the higher the probability of the peptide being bioactive.

### 2.8. Investigation of the Anti-Hypertension Effects of the Purified Peptide in Huvecs

#### 2.8.1. Cell Culture and Viability Assay

The experiments were carried out according to the method described by Yu-Mei et al. with slight modifications to protect the samples against damage to the HUVECs [20]. In the experimental group, 50 μL of CSPH2, CSPHF1 and CSPHF2 at 25, 50, 100, 500 and 1000 μmol/L were added to each well of the experimental medium, and the same volume of complete medium was added to each well. The supernatant was discarded, then 150 μL DMSO was added and shaken for 10 min. Control group: HUVECs were inoculated into 96-well plates containing 100 μL of medium and incubated for 24 h. After 24 h of incubation with PBS buffer solution (pH 7.2) instead of sample, 10 μL MTT was added to the plates and incubated for 4 h. The final mass concentration of each well was 200 μmol/L. The supernatant was aspirated, then 150 μL DMSO was added and shaken for 10 min. The absorbance of each group at 568 nm was measured according to Equation (4) to calculate the cell survival rate, and the sample concentration was selected according to the effect for the next experiment.

(4) $$\mathrm{Cell}\;\mathrm{viability}=\frac{{\mathrm{OD}}_{S}-{\mathrm{OD}}_{B}}{{\mathrm{OD}}_{C}-{\mathrm{OD}}_{B}}\times 100\%$$

OD_S_ was the absorbance of the sample, OD_B_ was the absorbance corrected for background absorption, and OD_C_ was the absorbance of the control absorption.

#### 2.8.2. Effects of the Purified Peptide on Huvecs

The method to establish the oxidative damage model of HUVECs referred to the report of Cai et al. [4]. A final concentration of 200 mM H_2_O_2_ was then chosen to model the oxidative damage to the HUVECs. The experiments were divided into the blank group, model group, positive control group and experimental group. In the blank group, PBS buffer solution (pH 7.2) was added to the medium and incubated for 24 h. Then, 10 μL MTT was added to the well plate and incubated for 4 h. The final mass concentration of each well was 200 μmol/L. In the model group, 200 mmol/L H_2_O_2_ was added to the medium instead of PBS buffer for oxidative damage treatment. In the positive control group, captopril was added as a positive control. In the model group, 200 mmol/L H_2_O_2_ was added to the medium instead of PBS buffer for oxidative damage treatment. In the positive control group, 160 μL of 100 μmol/L captopril was added to the model group for 24 h. Then, 10 μL of MTT was added to the well plate and incubated for 4 h. The final mass concentration of each well was 200 μmol/L. The supernatant was aspirated, and 150 μL DMSO was added and shaken for 10 min. Next, 160 μL of CSPH2, CSPHF1 and CSPHF2 were added to the experimental group instead of captopril at a concentration of 100 μmol/L, and the cell survival rate was calculated.

#### 2.8.3. Determination of the Content of NO

The assay was performed using the NO kit. Briefly, HUVEC cells were pre-incubated with concentrations of 100 μmol/L CSPH2, CSPHF1 and CSPHF2 for 12 h and then incubated with 200 μmol/L H_2_O_2_ for 2 h, respectively. Subsequently, groups of HUVECs were washed with PBS (pH 7.0, 0.02 mol/L) and cells were collected by centrifugation at 1000× *g* for 5 min; suspension cells could be collected directly by centrifugation. The collected cells were washed 3 times with PBS. For every 1 × 106 cells, 200 μL of PBS was added to resuspend and the cells were broken up by repeated freezing–thawing. The extracts were centrifuged at 3000 rpm (1006 g) for 10 min and the supernatant was removed for testing.

#### 2.8.4. Determination of the Content of ET-1

The ET-1 kit was used for the assay. The cell pre-treatment procedure was the same as in Section 2.8.3, and the supernatant was taken for the assay.

#### 2.8.5. Determination of Intracellular ROS Generation

The level of intracellular ROS was determined by a 2′,7′-dichlo-rofluorescein diacetate (DCFH-DA) fluorescent probe assay with an ROS Assay Kit. The cell pre-treatment procedure differed slightly from that in Section 2.8.3 in that 10 μmol/L DCFH2-DA was added to the PBS (pH7.0, 0.02 mol/L), followed by incubation for 0.5 h, and the supernatant was taken for testing.

### 2.9. Cellular Antioxidant Activities of CSPH

#### 2.9.1. Effect of CSPH on the Cell Culture and the Viability of HepG2 Cells

The incubator was set at 37 °C with 5% CO_2_, and HepG2 cells were grown in this environment, and the HepG2 cells were passaged every 2 days [21]. After 24 h of HepG2 culture, 100 μL of cell suspension was inoculated onto 96-well plates at a density of 1 × 104 cells/well, and then the cells were divided into control and experimental groups. The control group was prepared by adding 100 μL of medium and incubated for 24 h. In the experimental group, 100 μL of CSPH medium at concentrations of 50, 100, 200, 250, 500, 750, 1200 and 1500 μg/mL was added to each well, followed by the same volume of complete medium, and the incubation lasted for 24 h before adding MTT solution and incubating for 4 h. The final mass concentration of each well was 0.5 mg/mL, the supernatant was discarded, and 100 μL of DMSO was added to each well and shaken for 10 min. The cell viability was calculated by measuring the OD of the enzyme marker at 570 nm according to Equation (5).

(5) $$\mathrm{Cell}\;\mathrm{viability}\;\%=\frac{{\mathrm{OD}}_{1}-{\mathrm{OD}}_{2}}{\mathrm{Mean}\;{\mathrm{OD}}_{3}-{\mathrm{OD}}_{2}}\times 100\%$$

OD_1_ was the absorbance of the sample, OD_2_ was the absorbance corrected for background absorption, and OD_3_ was the absorbance of the control absorption.

#### 2.9.2. Establishment of H_2_O_2_ Oxidative Damage Model

The cell culture was performed according to the method in Section 2.9.1 and then the injury model group was established: 100 μL of H_2_O_2_ solution with concentrations of 0.1, 0.5, 1, 2, 5, 10, 20, 50 and 100 mmol/L was added to each well, and 6 parallel wells were set up for each well.

#### 2.9.3. The Protective Effect of CSPH on HepG2 Cells Damaged by H_2_O_2_

On the basis of the pre-experiments established by the above model, appropriate concentrations of H_2_O_2_ were selected and applied to the cells for 2 h, respectively. After determining the optimal damage conditions, the concentration that showed no significant toxic effect on cells was selected and tested as follows.

According to the experimental protocol of Wen et al. [22], the oxidative damage model selected a concentration of 5 mmol/L H_2_O_2_ and treated the cells for 2 h as the optimal oxidative damage condition. After the above two steps, 5 mmol/L of H_2_O_2_ and concentrations of 50 and 100 μg/mL of CSPH2–CSPHF1–2 were selected as the samples to study the protective effects on HepG2 cells, and the control, model and experimental groups were set up as follows. Control group: untreated with sample and H_2_O_2_; model group: treated with H_2_O_2_; experimental group: treated with samples CSPH2–CSPHF1–2 and H_2_O_2_, respectively.

To each well of the well plate, 100 μL of cell suspension at a density of 1 × 10^4^/mL was added and the medium was discarded after 24 h of incubation. In the control group, 100 μL/well of complete medium was added for 2 h. In the model group, 100 μL/well of complete medium containing 5 mmol/L H_2_O_2_ was added and incubated for 2 h. In the experimental group, 100 μL of CSPH2–CSPHF1–2 at concentrations of 50 and 100 μg/mL were added to each well, incubated for 24 h and then centrifuged. The supernatant was discarded and the MTT assay was performed after 2 h. The protective effect of the three samples on HepG2 cells was compared and the sample with the best effect was selected for the next experiment.

## 3. Statistical Analysis

Results are expressed as the mean ± standard deviation (n ≥ 3), and Duncan’s multiple range test was used for the variance analysis (*p* < 0.05). A one-way analysis of variance test for the differences between the means of each group was applied to analyze the data using SPSS 26.0 (SPSS Corporation, Chicago, IL, USA), and a *p*-value <0.05 was considered statistically significant. The Raw File of the mass spectrometry test was retrieved from the corresponding database using the software MaxQuant 1.5.5.1 to obtain the final protein identification and quantitative analysis results. Figures were drawn using Origin 2019b software (OriginLab Co., Northampton, MA, USA).

## 4. Results

### 4.1. Ultrafiltration and Bioactivity

**Figure 1:**
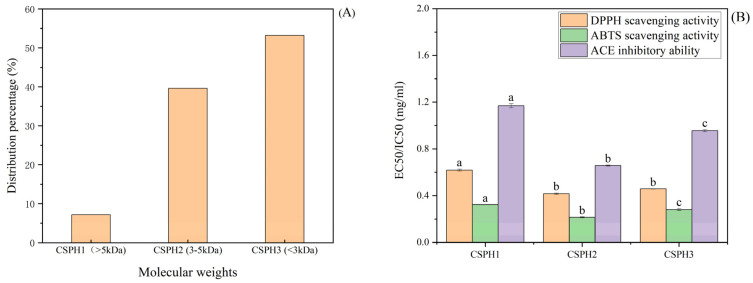
Distribution of different molecular weight peptides after ultrafiltration (**A**) and EC_50_/IC_50_ value (**B**) of hydrolysates. (Distinct letters denote statistically significant differences (*p* < 0.05) among molecular weight fractions within the same treatment group).

The enzymolysis products of a protein are a mixture of partially unhydrolyzed protein, polypeptides with different chain lengths and free amino acids, among others. As shown in Figure 1A, the CSPH was divided into three fractions according to the molecular weight by using the ultrafiltration membranes: CSPH1 (>5 kDa), CSPH2 (3–5 kDa) and CSPH3 (<3 kDa). The result from the gel permeation chromatography (GPC) showed that the majority of the peptides were made up of small oligopeptide compounds. CSPH3 took up the highest proportion 53.22%, followed by CSPH2 at 39.63%, and the lowest content was found for CSPH1 at 7.15%.

The scavenging activities of the DPPH free radical and ABTS were used to determine the antioxidant capacity. The EC_50_/IC_50_ of CSPH1, CSPH2 and CSPH3 are shown in Figure 1B. The scavenging activities revealed that the peptide enriched in CSPH2 possessed the best ability to scavenge free radicals among the three tested samples. It is worth noting that the EC_50_ of the component CSPH2 for the DPPH free radicals and ABTS radical cations was significantly lower than that of the CSPH1. Furthermore, the results of the ACE inhibition activity were consistent with the antioxidant capacity and revealed that the eluents with the component CSPH2 exhibited the best antioxidant activity and ACE inhibition activity and the lowest EC_50_/IC_50_ values. These results indicated that CSPH2 with molecules of 3–5 kDa was more active than the parts with larger or smaller molecular weights (*p* < 0.05).

### 4.2. Stability Analysis

**Figure 2:**
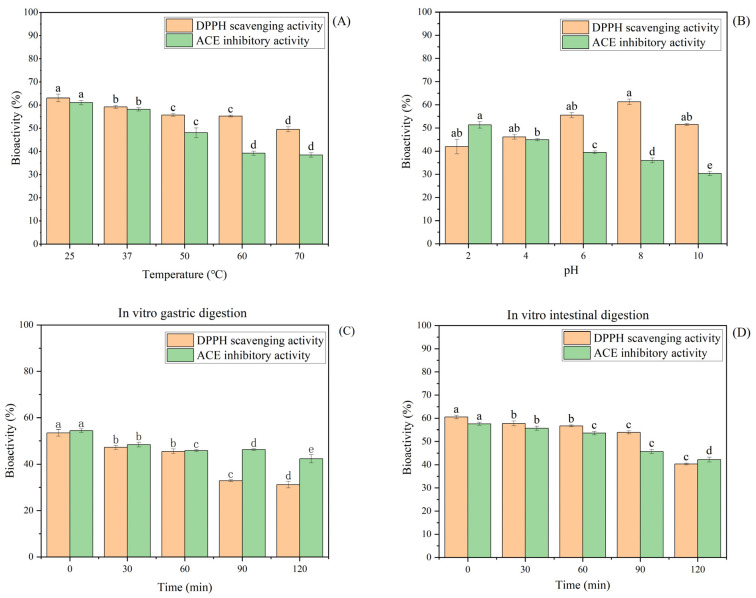
Effect of different factors on the stability of CSPH2: (**A**) temperature; (**B**) pH; (**C**) changes in the biological activity of CSPH2 after 120 min of in vitro gastric digestion; and (**D**) changes in the biological activity of CSPH2 after 120 min of in vitro intestinal digestion. (Different letters represent statistically significant differences (*p* < 0.05) among the effects of various factor levels under identical treatment conditions).

The stability of CSPH2 under different pH, temperature, and simulated gastrointestinal digestion conditions was investigated, and the results are presented in Figure 2. The DPPH scavenging activity of CSPH2 remained stable across the temperature range of 25–70 °C, with no significant changes observed. In contrast, the DPPH scavenging activity increased significantly as the pH was raised from 2 to 8. During in vitro simulated gastrointestinal digestion, the DPPH scavenging activity of CSPH2 remained stable during the middle gastric phase (30–60 min, *p* > 0.05) but declined significantly thereafter (*p* < 0.05 for >60 min). Meanwhile, at the early stage (<30 min), the DPPH scavenging activity also declined significantly. In artificial intestinal fluid, the antioxidant activity remained stable up to 60 min and then decreased sharply. Regarding the ACE inhibitory activity, a significant reduction was observed between 40 and 60 °C, after which the activity stabilized at higher temperatures. The ACE inhibitory activity also decreased significantly as the pH increased, indicating higher activity under acidic conditions. During simulated digestion, the ACE inhibitory activity remained unchanged between 30 and 90 min in gastric fluids but decreased after 60 min in artificial intestinal fluids.

### 4.3. Gel Filtration Chromatography of CSPH2

**Figure 3:**
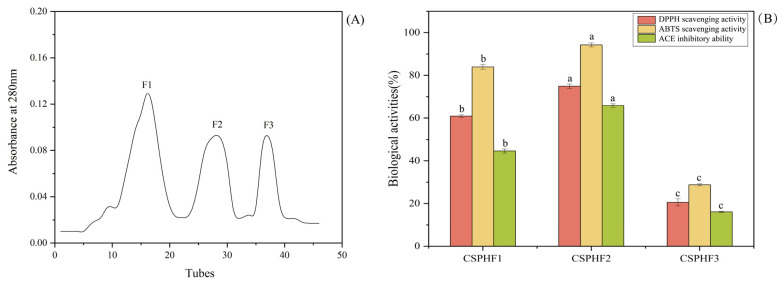
(**A**) Chromatogram of the graded fraction separated from CSPH2 by gel filtration chromatography. (**B**) Biological activity of the components. (Different letters indicate statistically significant differences (*p* < 0.05) in the biological activities of various components under identical treatment conditions).

CSPH2 was purified using a Sephadex G-25 column, resulting in three major absorbance peaks at 280 nm, as shown in Figure 3A. Three fractions (CSPHF1, CSPHF2, and CSPHF3) corresponding to these peaks were collected and lyophilized. The antioxidant activities (DPPH and ABTS assays) and ACE inhibitory activity of each fraction were determined at a concentration of 1.0 mg/mL, as shown in Figure 3B. Among the three fractions, CSPHF2 exhibited the highest DPPH scavenging activity (74.93 ± 1.03%), ABTS scavenging activity (94.26 ± 0.96%), and ACE inhibitory rate (65.88 ± 0.78%). CSPHF1 displayed moderate activity, while CSPHF3 showed the lowest bioactivity. Overall, both CSPHF1 and CSPHF2 demonstrated stronger antioxidant and ACE inhibitory activities than CSPHF3. Subsequently, LC-MS/MS was employed to identify the amino acid sequences of CSPH2, CSPHF2, and CSPHF3.

### 4.4. Identification of Antioxidant and Ace Inhibition Peptide

CSPH2, CSPHF1, and CSPHF2 were subjected to LC-MS/MS for peptide sequencing and identification of bioactive peptides. Peptides with PeptideRanker values greater than 0.85 and scores higher than 95 were selected. The mass spectrometry results of the crayfish shell protein products were analyzed by sequence information using the Mascot database and NCBI. The identified amino acid matching sequences from each sample are listed in Table 1. In total, 2, 7, and 10 bioactive peptides were identified in CSPH2, CSPHF1, and CSPHF2, respectively. The molecular weights of these peptides ranged from 800 to 1700 Da, with peptide lengths of between 8 and 18 amino acids. Notably, peptides with lengths ≤10 accounted for 52.63% of all identified peptides. Amino acid analysis showed that these peptides were rich in hydrophobic amino acids, such as valine, proline, alanine, and leucine/isoleucine. Several peptides contained repeating amino acid motifs, such as GPP and PPP. Some peptide sequences, such as PAPAPVPAPIFA and PAAPIPPAFN, had phenylalanine at the penultimate position, and a number of sequences contained proline, arginine, or lysine at the C-terminal position.

### 4.5. Cytotoxicity of CSPH2,CSPHF1 and CSPHF2 on Huvecs

**Figure 4:**
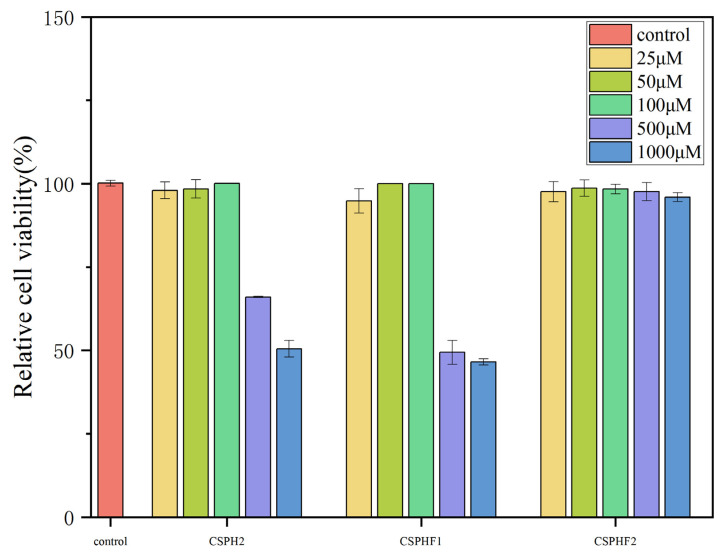
Effect of CSPH, CSPHF1, CSPHF2 on the viability of HUVECs.

To investigate the cellular mechanisms underlying the antihypertensive effects of the peptides, in vitro experiments were conducted using HUVECs. The cytotoxicity of CSPH2, CSPHF1, and CSPHF2 was evaluated by MTT assay. As shown in Figure 4, none of the three peptides exhibited significant cytotoxicity toward HUVECs at concentrations up to 100 μM (*p* < 0.05). At higher concentrations (500–1000 μM), CSPH2 and CSPHF1 displayed some cytotoxic effects, whereas CSPHF2 showed no or only low toxicity across all the tested concentrations. Among the concentrations tested, 100 μM yielded the highest cell viability for all three peptides; therefore, 100 μM was selected for subsequent experiments.

### 4.6. Effects of CSPH2, CSPHF1 and CSPHF2 on NO, ET-1, and ROS Scavenging in HUVECs

**Figure 5:**
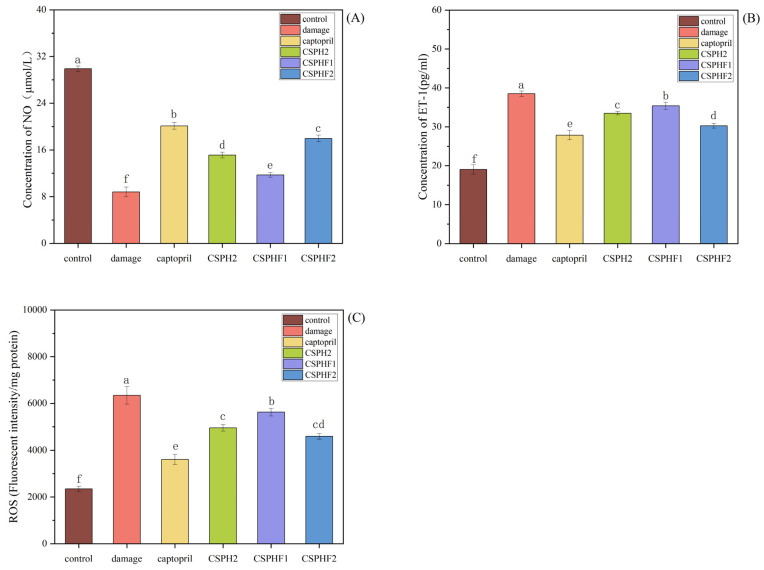
Effect of CSPH2, CSPHF1, and CSPHF2 on NO, EF-1 secretion and ROS scavenging in HUVECs: (**A**) NO; (**B**) ET-1; and (**C**) ROS. (Different letters indicate statistically significant differences (*p* < 0.05) among the components under identical treatment conditions).

The cellular mechanisms underlying the antihypertensive effects of the purified peptides were investigated in HUVECs by assessing their influence on NO and ET-1 secretion, as well as on ROS scavenging. As shown in Figure 5A,B, the levels of NO and ET-1 in the control group (without H_2_O_2_ treatment) were 20.11 ± 0.58 μmol/L and 27.86 ± 1.20 pg/mL, respectively. Following H_2_O_2_-induced damage, the NO levels decreased to 8.80 ± 0.83 μmol/L, while the ET-1 levels increased to 38.49 ± 0.68 pg/mL. Treatment with captopril significantly reversed these changes (*p* < 0.05), with the NO and ET-1 levels measured at 29.91 ± 0.48 μmol/L and 19.05 ± 1.20 pg/mL, respectively. All three peptide samples (CSPH2, CSPHF1, CSPHF2) promoted NO release and inhibited ET-1 secretion in damaged HUVECs. Notably, CSPHF2 increased NO from 8.80 ± 0.83 μmol/L to 17.96 ± 0.58 μmol/L and decreased ET-1 from 38.49 ± 0.68 pg/mL to 30.26 ± 0.61 pg/mL, achieving 80.99% and 77.42% of the effect of captopril, respectively.

Additionally, the ROS levels were evaluated (Figure 5C). H_2_O_2_ treatment significantly increased the ROS levels in HUVECs to 3608 ± 217/mg compared to 2349 ± 111/mg in the control group. Treatment with captopril reduced the ROS levels, and the three peptide samples also reduced the ROS production to 4951 ± 137/mg (CSPH2), 5624 ± 160/mg (CSPHF1), and 4590 ± 118/mg (CSPHF2), respectively. Among them, CSPHF2 showed the most pronounced ROS scavenging effect.

### 4.7. Establish of Oxidative Damaged Model of HepG2 Cells

**Figure 6:**
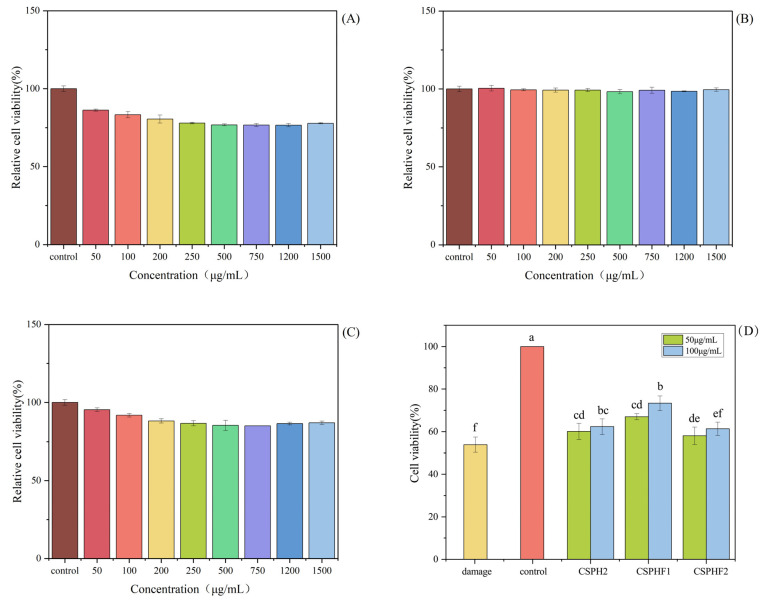
Effect of different concentrations of CSPH2, CSPHF1 and CSPHF2 on the relative viability of HepG2 cells: (**A**) CSPH2; (**B**) CSPHF1; (**C**) CSPHF2. (Different colors represent different concentrations of sample); and (**D**) comparison of cell protective effects. (Different letters indicate statistically significant differences (*p* < 0.05) among the components under identical treatment conditions).

As shown in Figure 6, no significant cytotoxic effects on HepG2 cells compared to the blank control (no peptide treatment) at concentrations of 50 and 100 µg/mL for 24 h treatment (*p* < 0.05) were observed. The result indicated that the three peptides, CSPH2, CSPHF1 and CSPHF2, from the protein hydrolysate of crayfish shell could serve as candidate molecules for antioxidant food and drugs. Moreover, 50 and 100 µg/mL were chosen as the mass concentrations of the experimental samples.

### 4.8. Cytoprotective Effects of Peptides on Oxidative Damaged Model of HepG2 Cells

Following oxidative stress, the balance of the endogenous antioxidant defense mechanisms was destroyed and failed to protect the cells from damage induced by oxidative stress, which finally led to significantly reduced cell viability. Then, antioxidant peptides were needed to protect the cells from oxidative stress. As shown in Figure 6, the H_2_O_2_ showed significant cytotoxic effects on HepG2 cells. H_2_O_2_ at a concentration of 5 mmol/L was used to establish the oxidative stress model of HepG2 cells, and the cell viability reached 53.92 ± 3.52% after being induced for 24 h in damage group.

## 5. Discussion

### 5.1. Purification, Identification and Digestive Stability of Csph

The size, composition, and amino acid sequence of the peptides are factors ultimately affect the biological activity of protein hydrolysates. A peptide fragment that is too long (more than 20 amino acids) often has a complex structure, in which the biologically active amino acid residues may be buried, resulting in a lack of functional activity. Small oligopeptides are easily absorbed by the body and can directly participate in the synthesis and metabolism of tissue protein. This is often assumed to be the reason for high biological activity. A peptide fragment of an appropriate length could show better functional activity [23].

Several studies have showed that peptides with lower molecular weight have higher antioxidant activity than those with higher molecular weights. However, the result was not in accordance with our study. This might due to the exposure of more hydrophobic residues when the crayfish shell protein molecule was enzymatically cleaved [24]. Although the molecular weight of CSPH2 was higher compared to CSPH3, it may contain more fragments of hydrophobic amino acids, which are closely associated with antioxidant properties and anti-ACE activity [25]. Therefore, CSPH2 was selected for further experiments.

The pH and thermal stability of peptides are critical factors in their production and processing, as well as key indicators when evaluating the effects of simulated processing treatments [26]. The results indicate that the antioxidant capacity of CSPH2 is not affected by temperature, suggesting robust thermal stability. The increased DPPH scavenging activity at higher pH levels may be attributed to the alkaline enzymatic hydrolysis used in the preparation of CSPH2, which potentially enhances its antioxidant properties under neutral to alkaline conditions. These findings are consistent with previous reports by Tavares et al. [27], demonstrating that certain peptides retain antioxidant activity following in vitro gastrointestinal digestion, particularly within specific time frames.

In contrast, the ACE inhibitory activity of CSPH2 was sensitive to temperature, showing a marked decline at elevated temperatures (40–60 °C), indicating that thermal processing may negatively impact its hypotensive potential. Moreover, the ACE inhibitory activity was higher in acidic conditions, which is advantageous for maintaining activity in the gastric environment; this observation aligns with findings by Lai et al. [26]. The relative stability of both the antioxidant and ACE inhibitory activities during the middle digestion times suggests good anti-digestibility, which is a desirable feature for bioactive peptides intended for functional food or therapeutic applications. Furthermore, the literature suggests that peptides containing proline residues are generally more resistant to proteolytic degradation [28], which could explain the observed stability of CSPH2 in the middle gastric phase of the simulated gastrointestinal fluids.Overall, CSPH2 demonstrates favorable stability profiles under various processing and physiological conditions, supporting its potential application as a functional food ingredient with antioxidant and ACE inhibitory activities.

The purification of CSPH2 by Sephadex G-25 chromatography effectively separated the hydrolysate into three fractions with distinct bioactivities. CSPHF2, in particular, displayed superior antioxidant and ACE inhibitory activities compared to the other fractions, indicating that the active peptides were primarily concentrated in this fraction. CSPHF1 also exhibited considerable bioactivity, whereas CSPHF3 showed minimal effects. These findings suggest that the bioactive components are not evenly distributed among the fractions and that purification can enhance the identification and utilization of potent peptide fractions. The application of LC-MS/MS for sequence identification provides a basis for further analysis of the structure–activity relationship of the peptides present in these fractions. Overall, the results support the potential of specific CSPH2-derived fractions as candidates for further development in functional food or nutraceutical applications.

The higher number of bioactive peptides identified in CSPHF2 compared to CSPH2 and CSPHF1 may explain the superior bioactivities observed in this fraction. Previous studies have indicated that peptides containing hydrophobic amino acids, such as His, Leu, Tyr, Met, Pro, Trp, Phe, and Val, tend to have stronger antioxidant activity [29]. The presence of repetitive motifs, especially GPP and PPP, as well as short peptide lengths, may contribute to enhanced antioxidant properties. It has also been reported that hydrophobicity, along with the type and position of amino acids, plays a key role in ACE inhibitory activity, particularly when aromatic amino acids are present at the penultimate position [30,31]. Furthermore, many ACE inhibitory peptides contain proline residues, especially at the N- or C-terminal, which is important for both activity and resistance to enzymatic degradation [32,33,34,35,36]. The structural motifs identified in this study, such as VPP and PLP, have previously been associated with ACE inhibitory and antihypertensive activities in vivo [37,38]. Shrimp shell samples with strong ACE inhibitory activities have been reported to be rich in phenylalanine, tyrosine, and proline at the C-terminal of peptides [39]. The position of AA in the peptide sequence also plays an important role in ACE inhibitory activity [40]. When the concentration of the peptide fraction was increased, an increase in inhibitory activity was noticed for CSPHF1 and CSPHF2. The features observed in the peptides in this study are consistent with bioactive peptides derived from other animal protein sources [36,41,42]. Therefore, the abundance and diversity of these structural motifs and amino acid compositions in CSPHF2 likely account for its greater bioactivity.

### 5.2. Anti-Hypertension and Anti-Oxidation Effects of the Purified Peptide on Huvecs and HEPG2 Cells

The results indicate that CSPH2, CSPHF1, and CSPHF2 are not cytotoxic to HUVECs at concentrations up to 100 μM, suggesting a favorable safety profile at physiologically relevant doses. However, at elevated concentrations, CSPH2 and CSPHF1 demonstrated increased cytotoxicity, whereas CSPHF2 maintained low toxicity even at higher concentrations. This difference in the cytotoxicity profiles may be related to differences in the peptide composition or sequence among the three fractions. The selection of 100 μM as the working concentration for further experiments was based on the observation that cell viability was optimal at this dose for all three peptides. These findings support the use of 100 μM as a safe and effective concentration for subsequent functional studies on the antihypertensive mechanism of the peptides in HUVECs.

The results demonstrated that the purified peptides, especially CSPHF2, exerted antihypertensive effects in HUVECs by modulating NO and ET-1 secretion and by reducing the ROS levels. ET-1 is recognized as a potent vasoconstrictor, and its overexpression is closely associated with the pathogenesis of cardiovascular diseases such as hypertension and atherosclerosis [43]. In contrast, NO functions as a vasodilator and can suppress ET-1 production, thus maintaining vascular homeostasis. The observed ability of the peptides to promote NO release and inhibit ET-1 secretion suggests a mechanism similar to that of captopril, which may involve inhibition of angiotensin II formation and activation of bradykinin receptors, leading to enhanced NO production and reduced ET-1 levels [44,45]. CSPHF2 exhibited effects closest to those of captopril, indicating its strong therapeutic potential as an antihypertensive agent.

Excessive ROS production is known to induce oxidative stress, contributing to the development of hypertension and related cardiovascular diseases [46]. All three peptides were able to reduce the intracellular ROS levels, with CSPHF2 having the greatest impact, suggesting its superior antioxidant capacity. The reduction of ROS may also imply that these peptides are capable of regulating apoptosis-related proteins, such as Bcl-2 family proteins, cytochrome C, and APAF-1, thereby offering protection against oxidative damage. The peptide sequences identified in this study contain several hydrophobic and aromatic amino acids, such as Pro, His, Val, Tyr, Met, and Leu, which are known to enhance both antioxidant and ACE inhibitory activities. The presence of hydrophobic amino acids enables effective binding to ACE catalytic sites, thus inhibiting enzyme activity [47]. Prior studies have shown that proline-rich sequences contribute significantly to antioxidant activity [48], and the occurrence of Val, Pro, and Leu in certain peptide motifs is associated with strong free radical scavenging effects [49]. Wang et al. found that antioxidant peptides from the collagen hydrolysate of redlip croaker could dose-dependently protect DNA in the oxidative damage HUVEC mode by the sequence of FPYLRH [50].

As with HepG2 cells, the cell viability of different concentrations of CSPH was lower than that of the control group (without H_2_O_2_ damage) but was significantly higher than that of the damage group, indicating that all three samples were able to effectively reduce the oxidative damage caused by H_2_O_2_ to the cells. The viability of the three sample-treated (CSPH2, CSPHF1 and CSPHF2) groups was gradually increased as the concentration increased compared with the damaged group. At a concentration of 100 µg/mL, CSPHF2 showed the strongest ability in terms of protecting HepG2 cell viability. It might due to the higher content of functionally active amino acids in CSPHF2, which therefore exhibited more pronounced bioactivity. Wu et al. found that in shrimp peptide, the Leu suppresses Bax, caspase-3, and p53 expression, as well as BCL-XL overexpression, thus protecting PC12 cells from oxidative stress. Both APAPLPPPAPVLPSPPR and APAPLPPPAP in CSPHF2 contained Leu, which might contribute to the high protection of the cell [51].

## 6. Conclusions

This is a detailed study on the antioxidant and ACE inhibitory peptides isolated from crayfish shell. Three peptide fractions (CSPH1–3) were isolated via the ultrafiltration membrane system, which exhibited strong antioxidant and ACE inhibitory activities. The fraction CSPH2 was the most potent antioxidant and ACE inhibitor. CSPH2 had good stability of gastric digestion and maintained high ACE inhibitory activity between 30 and 60 min and high antioxidant activity within 60 min. CSPH2 were further purified and three fractions were yielded, which were further identified by LC-MS/MS. The active structural domains APAPLPPPAP and QGPDDPLIPIM could explain the high antioxidant and ACE inhibitory activity of the crayfish shell peptide. Peptide fraction CSPHF2 presented the highest DPPH scavenging rate, ABTS scavenging activity and ACE inhibitor activity. The fraction was able to increase the NO levels and inhibit the secretion of ET-1 and ROS, indicating that crayfish-shell-derived peptide could serve as a promising candidate against oxidative stress and an antihypertensive drug for hypertension. The results indicated that the identified crustacean-derived peptides show the potential for developing functional food ingredients, dietary supplements, or nutraceuticals targeting specific health benefits. Nevertheless, this study focused solely on in vitro activity assessment of the peptides and their stability under simulated external temperature conditions, while lacking systematic in vivo validation as well as data on the peptide stability under food processing conditions and performance under real gastrointestinal conditions. Further research should focus on conducting in vivo studies to validate the observed bioactivities and assess the bioavailability and safety in relevant animal models. Future studies should investigate the properties and efficacy of these peptides within specific food matrices or supplement formulations under realistic processing and storage conditions.

## Figures and Tables

**Table 1:** CSPH2, CSPHF1 and CSPHF2 were separated and the peptides were identified by MS.

Sample	Sequence	MW	Sequence Length	Protein Accession IDs	Score
CSPH2	QGPDDPLIPIM	1194.5955	11	QGC85410.1	117.4
	APAPLPPPAP	926.52255	10	XP_045598893.1	100
CSPHF1	MPPPVPPF	880.4517	8	XP_045590478.1	114.5
	YGPPPPGPPPAPPMR	1526.7704	15	XP_045600860.1	117.31
	LPPLEPFPF	1055.5692	9	XP_045612887.1	103
	SPFDKPGPPIGPF	1354.6921	13	XP_045612887.1	1283
	APAPLPPPAPVLPSPPR	1672.9665	17	XP_045598893.1	158.3
	APAPLPPPAP	926.52255	10	XP_045598893.1	100
	PAAPIPPAFN	993.52837	10	XP_045618911.1	105.3
CSPHF2	GPPGGPPGGPPGPPPFGR	1593.8052	18	XP_045605857.1	2383
	MPPPVPPF	880.4517	8	XP_045590478.1	134
	YGPPPPGPPPAPPMR	1526.7704	15	XP_045600860.1	117
	QGPDDPLIPIM	1194.5955	11	QGC85410.1	117
	APAPLPPPAPVLPSPPR	1672.9665	17	XP_045598893.1	158
	APAPLPPPAP	926.52255	10	XP_045598893.1	100
	PAAPIPPAFN	993.52837	10	XP_045618911.1	105
	PAPAPVPAPIFA	1146.6437	12	XP_045620911.1	100
	HAPVFPGAPF	1038.5287	10	XP_045592091.1	110
	APHDPIVFPR	1147.6138	10	XP_045613622.1	98

## Data Availability

Data will be made available on request.
